# The Functional Role of the Triceps Surae Muscle during Human Locomotion

**DOI:** 10.1371/journal.pone.0052943

**Published:** 2013-01-16

**Authors:** Jean-Louis Honeine, Marco Schieppati, Olivier Gagey, Manh-Cuong Do

**Affiliations:** 1 CIAMS Laboratory, UFR STAPS, University Paris-Sud, Orsay, France; 2 CSAM Laboratory, Salvatore Maugeri Foundation (IRCCS) and University of Pavia, Pavia, Italy; 3 Department of Orthopaedics, Faculty of Medicine, University Paris-Sud, Le Kremlin-Bicêtre, France; Dalhousie University, Canada

## Abstract

**Aim:**

Despite numerous studies addressing the issue, it remains unclear whether the triceps surae muscle group generates forward propulsive force during gait, commonly identified as ‘push-off’. In order to challenge the push-off postulate, one must probe the effect of varying the propulsive force while annulling the effect of the progression velocity. This can be obtained by adding a load to the subject while maintaining the same progression velocity.

**Methods:**

Ten healthy subjects initiated gait in both unloaded and loaded conditions (about 30% of body weight attached at abdominal level), for two walking velocities, spontaneous and fast. Ground reaction force and EMG activity of soleus and gastrocnemius medialis and lateralis muscles of the stance leg were recorded. Centre of mass velocity and position, centre of pressure position, and disequilibrium torque were calculated.

**Results:**

At spontaneous velocity, adding the load increased disequilibrium torque and propulsive force. However, load had no effect on the vertical braking force or amplitude of triceps activity. At fast progression velocity, disequilibrium torque, vertical braking force and triceps EMG increased with respect to spontaneous velocity. Still, adding the load did not further increase braking force or EMG.

**Conclusions:**

Triceps surae is not responsible for the generation of propulsive force but is merely supporting the body during walking and restraining it from falling. By controlling the disequilibrium torque, however, triceps can affect the propulsive force through the exchange of potential into kinetic energy.

## Introduction

Putting in motion any material system requires the application of external forces. Bipedal locomotion is no exception. In human walking, forces are necessary for propelling the body forward. At the same time, due to the gravitational attraction exerted by the Earth, upward-directed forces are obligatory for keeping the body in equilibrium and preventing it from falling. Plantar flexor muscles are good candidates for generating both forces, since they exert their action at the interface between the human body and the ground. There is no general consensus as to whether the triceps surae muscle, the major plantar flexor group, contributes to the propulsive action (active thrust or ‘push-off’) or to the support action, or both.

Debate has been surrounding the functional role of ankle flexors for some time. Originally, based on the existing co-variation between triceps surae electromyographic (EMG) activity and velocity of progression, Winter [1] suggested that ankle plantar flexors provide the active push-off during the late part of the single stance phase. In contrast, Perry [2] had advised dropping the term push-off and postulated that the peak of the ground-reaction force in late stance phase is the result of the leverage put forth by the body alignment with respect to Earth-vertical axis rather than of an active downward thrust. An introduction to these issues can be found in an influential paper by Sutherland *et al.* [3]. At the other temporal edge, Bogey *et al.* [4] have provided an extensive review of more recent reports, to which the reader is referred.

The push-off hypothesis has been endorsed by a series of articles modelling the net ankle moment [5], [6], [7], [8], [9]. However, other experiments have provided results that undermine this hypothesis. Tibial nerve block (with paralysis of triceps surae muscle along with plantaris, tibialis posterior, flexor hallucis longus, and flexor digitorum longus) resulted in an *increase* in forward velocity of the centre of mass (CoM) during the late stance phase of gait, which was interpreted as defective control of CoM fall [3], [10]. Replacement of one lower limb by a prosthesis did not affect the speed of progression, regardless of whether the stance limb was prosthetic or not [11]. These findings support Perry’s [2] statement and the conclusion of a seminal paper by Cavagna and Franzetti [12], who posited that the fall of the CoM during the single stance phase of gait is enough for transforming the potential energy into forward kinetic energy for progression during normal level walking. Therefore, triceps surae would not provide the propelling thrust by pushing off the ground. Hence, the contribution of the plantar flexors to whole-body forward displacement during normal walking would primarily consist in restraining forward tibial rotation, thereby stabilising the knee joint [3], and in controlling the braking of the fall of the CoM during the single support phase of gait, as recently suggested by Chastan *et al.* [13].

The role of the ankle flexors in body support during gait has been agreed upon in the literature. The postural role of soleus in quiet standing is amplified during locomotion [14], [15], which imposes more body support as ankle torque increases [16], [17]. Anderson and Pandy [18] studied the muscle contribution to body support using mathematical modelling based on a force-sharing problem algorithm. They found that the ankle flexors generated nearly all the body support in early and late stance, in addition of being responsible for the second peak typically observed in the vertical ground reaction force. Neptune *et al.* [6] used a more elaborate model based on forward dynamics, and stated that the gastrocnemii and soleus provide trunk support during single stance and pre-swing. They stressed however the fact that in late stance phase the energy produced by the soleus accelerates the trunk forward, while the gastrocnemii would deliver most of their energy to accelerate leg into swing. On the other hand, Liu *et al.* [19] used mathematical modelling to simulate locomotion at varying walking velocities, and concluded that an increase in gastrocnemii and soleus activity accompanies body support as walking speed increases.

Our aim was to unravel the functional role of ankle plantar flexors during human locomotion. We set out to see whether the triceps surae provides forward thrust by pushing off the ground or it controls body dynamic equilibrium, or does both at the same time. The problem here lies in the fact that the coordination and synergy produced by the walking body make that several gait parameters are highly correlated. Propulsive force, forward velocity and triceps surae EMG during the stance phase are one example [1], [20]. We posited that in order to properly assess the role of ankle plantar flexors in gait, one should *increase* propulsive forces for a *constant* forward velocity. This can be done by adding an extra load to a subject and instruct him/her to maintain constant walking velocity. Since adding a load requires greater external force to move the body, then an increase in plantar flexor EMG activity for the greater antero-posterior force at the same walking velocity would verify the push-off hypothesis. Conversely, the absence of load-related increase in plantar flexor activity would discard the push-off hypothesis and support a functional role of these muscles in balance control.

The gait initiation paradigm was used [21], [22], [23]. This choice was based on the fact that subjects initiating walking from a static upright position, when the initial forward-directed velocity is null, would actively produce the whole of the propulsive force according to the push-off concept. The push off might be less necessary if gait was already in the stationary state, when the body speed itself would produce part of the propulsive force. So, failure to support the push-off hypothesis during gait initiation would reinforce with stronger reason the alternative hypothesis. We concurrently analysed the contribution of gravity in creating propulsive force by measuring the displacement of the CoM away from the support axis, which generates the forward disequilibrium torque. Two walking velocities were tested, in order to generalize the main conclusion to different progression speeds, in which neural mechanisms such as altered fusimotor drive, reduced pre-synaptic inhibition and/or increased descending excitatory input may undergo subtle changes [24].

## Materials and Methods

### Subjects

Ten healthy volunteers (one female and nine males) took part in the experiment. Their mean age, body mass and height were 34 years (range 23–54), 72 kg (61–83) and 1.73 m (1.69–1.83), respectively. Written informed consent was obtained, as required by the Declaration of Helsinki and by the EA 4532 local Ethics Committee of University Paris-Sud, who specifically approved this study.

### Experimental Set-up

A large force platform (0.90 m×1.80 m, AMTI, USA) was used to record ground reaction force and moments. The force platform was embedded in the ground. The walkway was long enough (7 meters) to allow subjects to carry out at least 6 steps, hence avoiding the interference of gait termination with the gait initiation motor programme [25].

The overall mass of the added load was 20 kg and consisted of 2 weight-lifting disks of equal mass and size (29 cm diameter and 3 cm thickness) put in two backpacks carried by the subject at the abdominal and lumbar back level, i.e. roughly around the centre of mass (CoM) position. The backpacks were firmly wrapped to the body to avoid unwanted displacement during stepping. For a subject weighting 83 kg, a total mass of 30 kg load was used instead. So the added load increased the body weight by a proportion ranging from 25% to 33%, depending on the subject.

Surface EMG activity was recorded using bipolar Ag-AgCl electrodes (8 mm diameter, 20 mm inter-electrode distance). Electrode sites were prepared by cleansing and shaving the skin for optimal myoelectric impedance. EMG activity was collected from right and left tibialis anterior (TA), soleus (SOL), gastrocnemius medialis (GM) and gastronemius lateralis (GL) muscles by preamplified wireless electrodes (Zero-wire, Aurion, Milan, Italy). GM and GL were recorded from only 7 of the 10 subjects. EMG signals were amplified (x1000) and band-pass filtered (10–500 Hz). Electrodes were placed according to the SENIAM protocol [26]. Force platform and EMG data were digitised at a sampling frequency of 1000 Hz on the same A/D converter card and saved on a PC for off-line analysis.

### Experimental Protocol

Before recording, we determined the preferential starting foot of the subjects. Subjects were asked to stand still eyes closed, and a small thrust was applied to their back forcing them to make a step forward. This was repeated 3 times. Then, subjects were instructed to initiate gait with the stepping leg that was used during this test. In order to obtain a good reproducibility of the progression velocity during the experiment, subjects executed several blank trials to determine the steps lengths corresponding to their spontaneous (S) and fast (F) speed walking conditions, and two landmarks representing the step length for the S and F condition were then drawn on the force platform for each subject. The average walking velocity proved to be about 1.1 ms^−1^ at spontaneous and 1.5 ms^−1^ at fast velocity (see Results).

Subjects stood still, barefoot on the force platform, looking straight ahead, and initiated gait following a verbal go-signal. They were instructed not to start walking in a reaction-time mode, but to start when they felt ready. This usually occurred within 2s from the go-signal. Both S and F conditions were repeated with and without the added load (L). The four experimental conditions are termed S, S+L, F and F+L in the text. All subjects started by performing the spontaneous unloaded series, following which the other conditions were performed in a pseudo-random order. Fifteen trials were acquired in each experimental condition.

### Ground Reaction Force and Disequilibrium Torque

**Figure 1:**
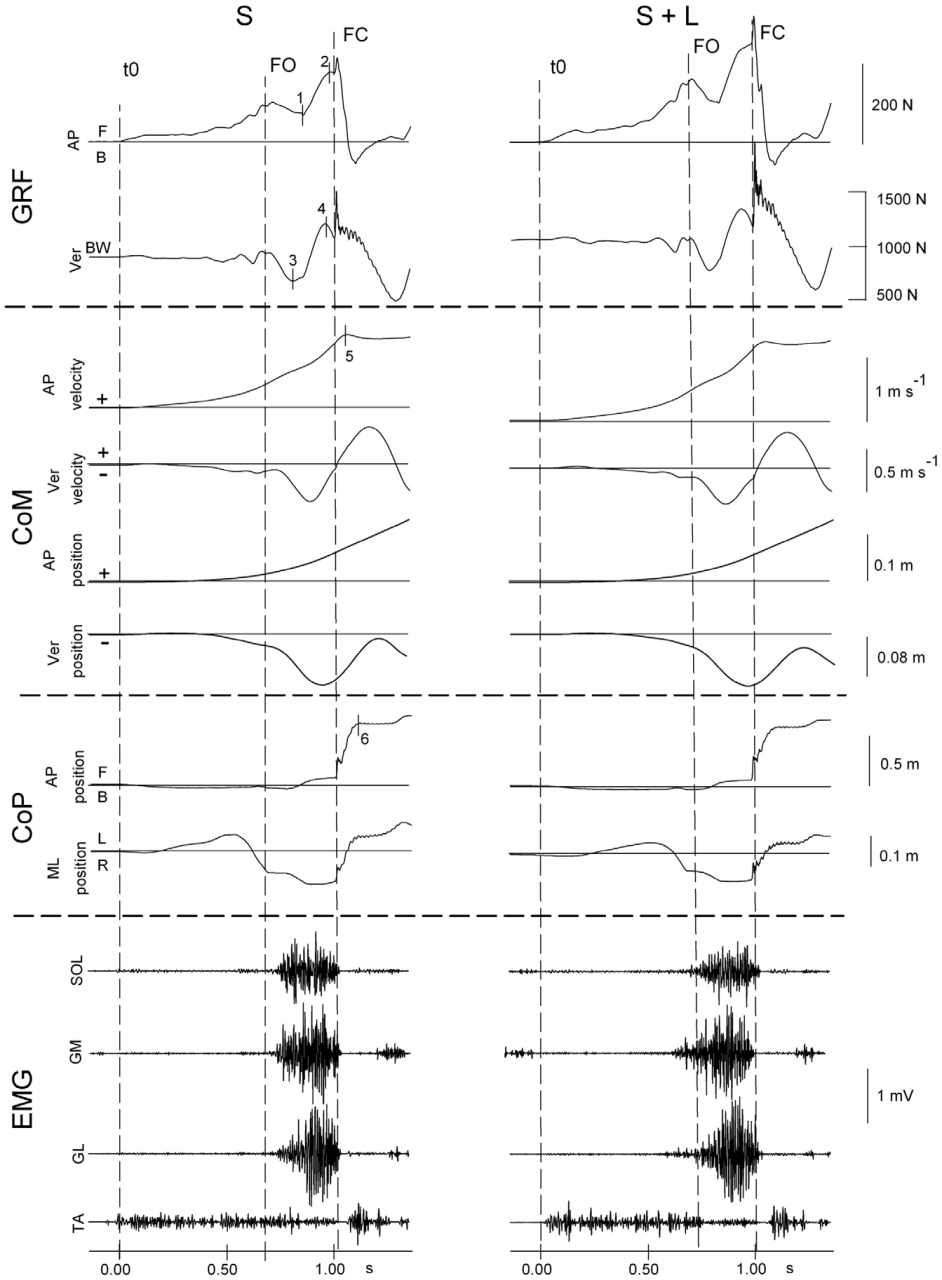
Time-course of gait initiation variables in a representative subject. All mechanical and EMG traces refer to walking at spontaneous (S) velocity. The left panel shows the control condition (no added load), the right panel shows the loaded (L) condition. The traces are assembled in four panels according to the type of recording. From top to bottom: Ground Reaction Forces (antero-posterior and vertical GRF), Centre of Mass (antero-posterior and vertical velocity and position of CoM), Centre of Foot Pressure (antero-posterior and medio-lateral CoP), EMG activity of the triceps surae muscles of the stance leg (soleus, SOL; gastrocnemius medialis, GM; gastrocnemius lateralis, GL; tibialis anterior, TA). The direction of the changes in the antero-posterior (AP) and medio-lateral (ML) position of GRF and CoP is indicated (forwards, F; backwards, B; left, L; right, R). The signs+and – in the CoM traces refer to positive and negative values of CoM velocity and position in the AP and vertical (Ver) direction. All traces start at time 0, corresponding to the onset of the anticipatory adjustment preceding the production of the first step, based on the (magnified) trace of the ML CoP position. The vertical dotted lines are set at the instant of foot off (FO) and foot contact (FC) of the swinging leg. The period between FO and FC is the single stance phase of gait. The triceps muscles are active during this phase, starting shortly after FO and terminating around FC. The numbers and ticks on selected traces of the left panel indicate critical points for the analysis: 1–2, onset and offset of the propulsive force increase; 3–4, onset and offset of the braking action; 5, peak of AP CoM velocity; 6, AP CoP position used to determine step length. Adding the load (right panel) increases Ver GRF, the value of which corresponds to the body weight (BW) in the period from t0 to FO. The load also increases the difference (2-1) of the AP component of the GRF, but has negligible effect on the other variables. Notably, adding the load has no effect on the ‘braking action’, or the difference (4-3) in the Ver component of the GRF, and in the pattern of triceps activity.

While walking, the body exerts a force on the ground, which in return applies on the subject an opposite force, the ground reaction force (GRF) that is measured by the force platform. This force was divided into 3 components (antero-posterior, AP; medio-lateral, ML; vertical, Ver). In addition, the force platform gave two moments with respect to the AP and ML axis of the platform. The coordinates of centre of pressure (CoP) were obtained by dividing these moments by the vertical GRF. The CoP instantaneous position was used to establish the instant of foot off (FO) and foot contact (FC). FO was the point at which the ML CoP position shifts under the stance foot, and FC the point at which the AP CoP position suddenly increases as the swing foot touches the ground (see Fig. 1, 7^th^ & 8^th^ trace). CoM velocity was obtained by integration of the CoM acceleration. CoM forward acceleration was obtained as AP GRF/BM, and CoM vertical acceleration as (Ver GRF-BW)/BM, where BW and BM are body weight and body mass, respectively. The instantaneous position of the CoM was obtained by double integration of the CoM acceleration with respect to time [13], [27].

During gait, CoM oscillates vertically while rotating around the CoP in the sagittal plane, and the CoM fall is braked during the single stance phase of gait. The displacement of the CoM generates a disequilibrium torque, driven by gravity, as CoM moves beyond the CoP. The braking of the CoM fall was evaluated as the variation of the vertical GRF between its minimum and maximum value within the single stance phase (points 4 and 5 in Fig. 1). The disequilibrium torque was calculated as Ver GRF·(CoM-CoP). The difference (CoM-CoP) represents the instantaneous distance (hereafter called gap) between the AP position of the CoP and the corresponding position of the ground projection of the CoM.

### EMG Analysis

The EMG activity of SOL, GM and GL was rectified after removing the baseline offset and was time-integrated. EMG activity of each muscle was calculated for three partly overlapping time windows: Wtot corresponds to the total duration of the burst, between the onset and termination of the EMG activity; Wb is the braking action phase and Wp is the propulsive phase. The braking action goes from the instant when Ver GRF reverts and goes upwards until the time of foot contact (FC), and the propulsive phase corresponds to the time interval initiating at the instant when AP GRF increases steeply and terminating just prior to FC (points 1 and 2 in Fig. 1). The integrated EMG activity of each trial was then divided by the time duration of each window in order to get the mean activity level. For graphical representation, EMG activity was expressed as a percentage relative to the mean value of the unloaded spontaneous velocity condition for each muscle and for each of the three time-windows.

### Statistical Analysis

The focus of the present study was the effect of load on muscle activity for a same walking velocity as opposed to the effect of velocity itself. However, velocity was selected as independent variable in order to check the statistical difference between the velocities. Two-way ANOVA was used to compare each of the measured or calculated biomechanical variables. Categorical factors were velocity and load. The mechanics-related variables were: forward velocity, step length, instants of occurrence of FO (foot-off of the swinging leg) and FC (foot contact of the same leg), amplitude of the sudden increase of the propulsive force (the ‘push-off’) and amplitude of vertical braking action. The EMG-related variables were the instants of onset and offset of the activity of each of the muscles with respect to time of gait initiation, and the mean level of the EMG surface of each muscle for each of the time windows (Wtot, Wp and Wb). Paired t-test was used to test the effect of load on the delay between the onset of SOL and the onset of the braking action. The level of significance was set at p<0.05 for all tests.

## Results

In Fig. 1, mechanical and EMG traces for the S condition (spontaneous velocity, unloaded condition) are compared with the S+L (spontaneous velocity, loaded condition). The overall kinematics and lower limb muscle activity of the gait initiation process have been described in detail elsewhere [21], [23], [27]. Briefly, the gait initiation process includes two phases. The former is an anticipatory postural adjustment (APA), which starts at the onset of the ground reaction force (GRF) variation (t0 in Fig. 1) and lasts until the first foot-off (FO). The APA prepares the step execution by means of a motor programme involving a deactivation of SOL background EMG activity, followed by a bilateral TA activation. Both events produce the backward displacement of the CoP. This produces a gap between the CoP position and the vertical projection of the CoM, causing a forward disequilibrium torque [23], [28]. The latter phase is the step execution that follows the APA. It goes from the time of FO of the swinging leg until the foot-contact (FC) of the same leg.

Kinematics results (Table 1) show that all subjects faithfully executed the experimental instruction, and thus maintained their walking velocity and step length regardless of the added load, thereby producing the same velocity condition. For the progression velocity, the grand mean value was almost identical without and with load for the spontaneous velocity. The load had no effect on the progression velocity for the fast velocity, either. Adding the load had no effect on step length, for either the spontaneous or fast velocity conditions. The instants of FO and FC with respect to t0 did not change with added load, either, and this was true within the spontaneous and the fast velocity conditions (Table 1).

**Table 1:** Kinematic Variables.

		FO (s)	FC (s)	AP Vel (m·s^−1^)	Step length (m)
N	mean ± SD	0.571±0.05	0.955±0.06	1.09±0.07	0.59±0.03
N+20	mean ± SD	0.605±0.05	0.957±0.06	1.09±0.07	0.61±0.03
	F (1,9); P	2.7; 0.13	0.003; 0.95	0.007; 0.93	3.3; 0.10
F	mean ± SD	0.597±0.06	0.968±0.05	1.56±0.07	0.84±0.03
F+20	mean ± SD	0.609±0.07	0.988±0.06	1.517±0.08	0.85±0.03
	F (1,9); P	0.2; 0.63	0.6; 0.44	1.1; 0.31	0.2; 0.69

Grand mean and standard deviation of the instant of foot off (FO) and foot contact (FC) of the swing leg with respect to the onset of movement (t0), the peak antero-posterior CoM velocity (AP Vel) (shortly after FC), and the step length calculated as the difference of the AP position of CoP between the end and the beginning of the stance phase. F and p values are reported to show that load had no significant effect on any of these kinematic variables for both velocity conditions.

### Spontaneous Velocity, Unloaded and Loaded Condition

During single stance (FO to FC in Fig. 1), the AP GRF (top trace) had a two-phase time-course, describing an early small variation followed by a steep increase (the propulsive phase). The onset of this increase (point 1) was set at the minimal value of AP GRF during the single stance phase that occurred usually around mid-stance. The magnitude of the increase is the difference in AP GRF between points 1 and 2.

Ver GRF trace was valley-shaped. Shortly after FO, Ver GRF started decreasing below subject’s body weight, reflecting CoM downward acceleration. After attaining a minimum value (point 3), it increased again and reached a value well beyond body weight (point 4). The variation of Ver GRF between points 3 and 4 is the braking action, or the vertical force opposing the CoM fall seen in the vertical CoM velocity trace.

Visual inspection of the two columns of Fig. 1 (left, S; right, S+L) shows that the difference in the amplitude of AP GRF between point 1 and 2 increased with the added load. Conversely, adding the load did not obviously change the braking action amplitude.

The onset of the increase in Ver GRF always preceded the onset of the steep increase in AP GRF, while the duration of the braking action and that of the propulsive phase overlapped for a while during the single stance period (from FO to FC). More precisely, the time window of the braking action phase accounted for 66% ±10, 64% ±4, 66% ±5 and 63% ±6 of the entire single-stance duration made equal to 100% for S, S+L, F and F+L conditions, respectively. The time window of the propulsive phase accounted for 41% ±14, 43% ±8, 49% ±7 and 55% ±8 of the single stance duration for S, S+L, F and F+L conditions, respectively.

Velocity and position of the CoM are shown in Fig. 1, below the GRF traces. The peak of AP CoM velocity was reached after FC (3^rd^ trace from top, point 5). The negative value of the vertical CoM velocity until FC indicates that the CoM was always falling during the whole single support period, but with two phases, the first accelerating downward, the second decelerating, when the fall of CoM was being restrained. The Ver CoM instantaneous position (6^th^ trace from top) fell below its initial value during the single stance, stabilised slightly around foot contact and moved up again during the double support phase while the triceps surae muscles were being deactivated. The CoM curve matches well in profile and maximum value the curve calculated with an independent method (a motion capture system) by Jian *et al.* [29]. Multiplying the gap between the CoM and CoP by the vertical force acting on the CoM (Ver GRF) gave the disequilibrium torque acting around the CoP.

The third panel from top shows the CoP traces. The CoP underwent a displacement from the rear to the fore foot, where its forward movement was obviously halted due to foot length limitation, while the CoM continued to advance on the sagittal plane in a parabolic manner (5^th^ trace from top in Fig. 1).

Fig. 1 (bottom traces) also illustrates the SOL, GM, GL and TA EMG activity of the stance leg. Triceps surae muscles were silent during the postural adjustment phase, while their synchronous bursts preceded shortly the vertical braking action and ended at around FC. This was true under both unloaded and loaded conditions. The onset of the burst occurred in the interval between FO and the onset of the braking action and varied slightly between subjects and repeated measures. One source of variability between trials likely depended on the force platform registering the global resultant forces and not the local forces produced by the individual muscles. This variation also affected the onset and termination of the muscle bursts. Another source of variability depended on the way the onset of the braking action was identified, since this was conservatively set at the lowermost point of the Ver GRF trace, in a region where the profile is not particularly sharp.

**Figure 2:**
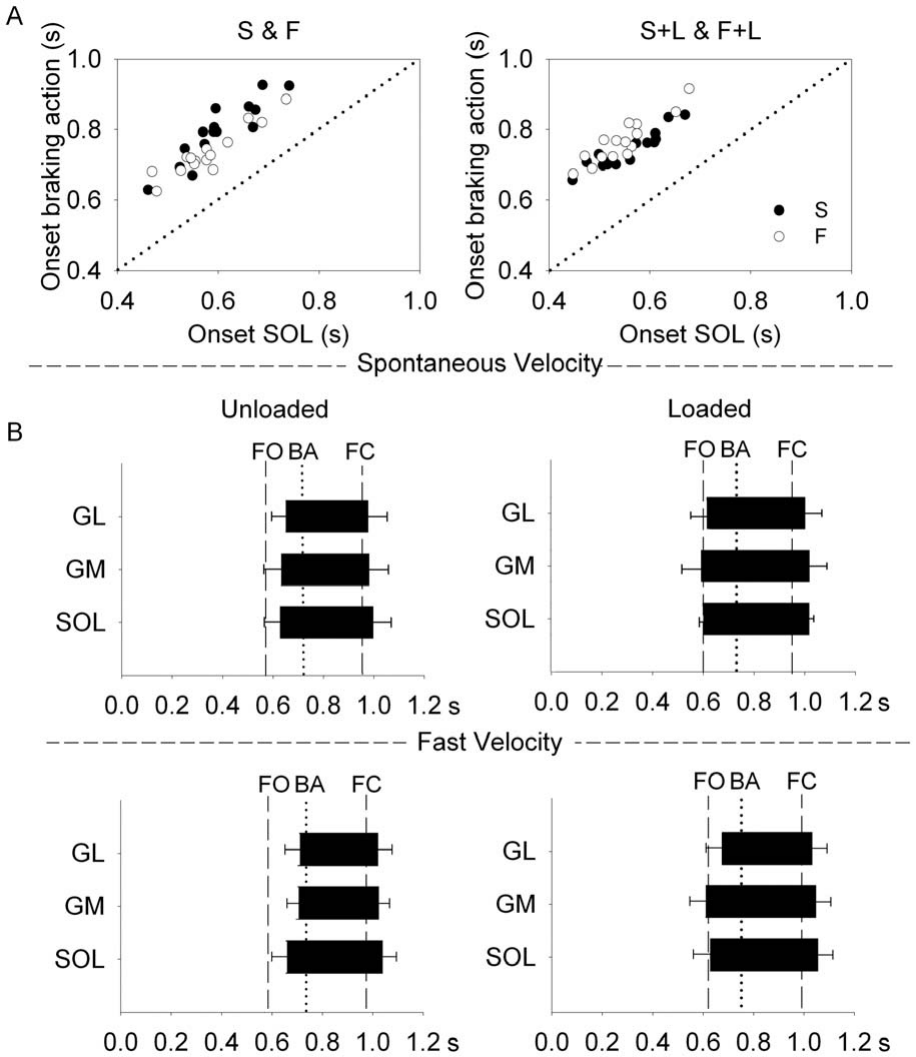
Temporal pattern of activity of the triceps surae muscles (SOL, GL, GM) during the stance phase of gait initiation. (A). The onset of the braking action has been plotted as a function of the onset of the SOL EMG activity for the unloaded (left) and loaded condition. The individual data points corresponding to spontaneous and fast velocities are superimposed in each plot. The braking action regularly lags the onset of SOL activity, so that the data points identify a line parallel to the identity line (dotted diagonal). In this subject, the points for spontaneous and fast velocity are almost confounded, and the points for the loaded condition indicate a small delay of the onset of the braking action with respect to the onset of the muscle activity. Such behaviour is only in part reflected in the other subjects, so that the mean intercept of the best fit lines are not significantly different between unloaded and loaded conditions. (B). The grand mean values (± SD) of onset and termination of the bursts of activity are reported for the three triceps muscles, referred to time 0 for all subjects and conditions of load and velocity. On the same time scale, the mean instants of foot-off (FO) and foot contact (FC) of the swing leg are indicated by vertical dashed lines. The vertical dotted lines refer to the mean onset of the braking action. The two top graphs refer to spontaneous (S) walking velocity, unloaded (left) and loaded (right). The data of the fast (F) velocity conditions are reported in the bottom graphs. There is no clear-cut difference neither in the overall time pattern of the activity across muscles, nor between S and F or between S+L and F+L. However, for both velocities, load increased the duration of the bursts, chiefly by anticipating the onset of their activity with respect to FO.

In spite of these sources of variations, consistent findings were observed in the EMG pattern. On the average, all muscles initiated their activity around FO, and ahead of point 4 (the onset of the braking action), and terminated at or shortly after FC. Fig. 2 A shows the onset of the braking action plotted versus the onset of the SOL activity, both measured with respect to FO, under both unloaded and loaded conditions for one subject. In this example, but also for all muscles and subjects, the braking action never anticipated the EMG onset. The onset of SOL activity with respect to FO was significantly anticipated in the loaded trials (F(1,9) = 5.183, p<0.05); however velocity *per se* had no significant effect (F(1,9) = 0.01, p = 0.92). The grand mean and standard deviation of the time-interval between onset of SOL and onset of braking action were 0.124±0.06 s for the unloaded trials and 0.199±0.05 s for the loaded trials. A paired t-test showed that load significantly increases the delay between the onset of SOL and that of breaking action (p<0.001).

The histogram bars of Fig. 2 B show the time-course of the EMG activity of the soleus and gastrocnemii muscles of the stance leg (with reference to FO and FC, respectively), for spontaneous and fast velocity, and for unloaded and loaded condition. The time-intervals between onset of EMG and onset of braking action (BA, dotted line) are broadly superimposed for the different muscles, with some anticipation for the SOL, inconsistent across trials and subjects. To note is an advancement of the onset of the EMG bursts with load for both velocities, in the face of a substantial similarity in the time distribution of the EMGs between spontaneous and fast velocity. ANOVA showed no significant difference in the time of onset across the three muscle bursts (SOL, GM and GL) (F(2,6) = 0.96, p = 0.40), indicating a concurrent recruitment and a common action of the triceps surae group aimed to brake the fall of the body during the single stance phase.

**Figure 3:**
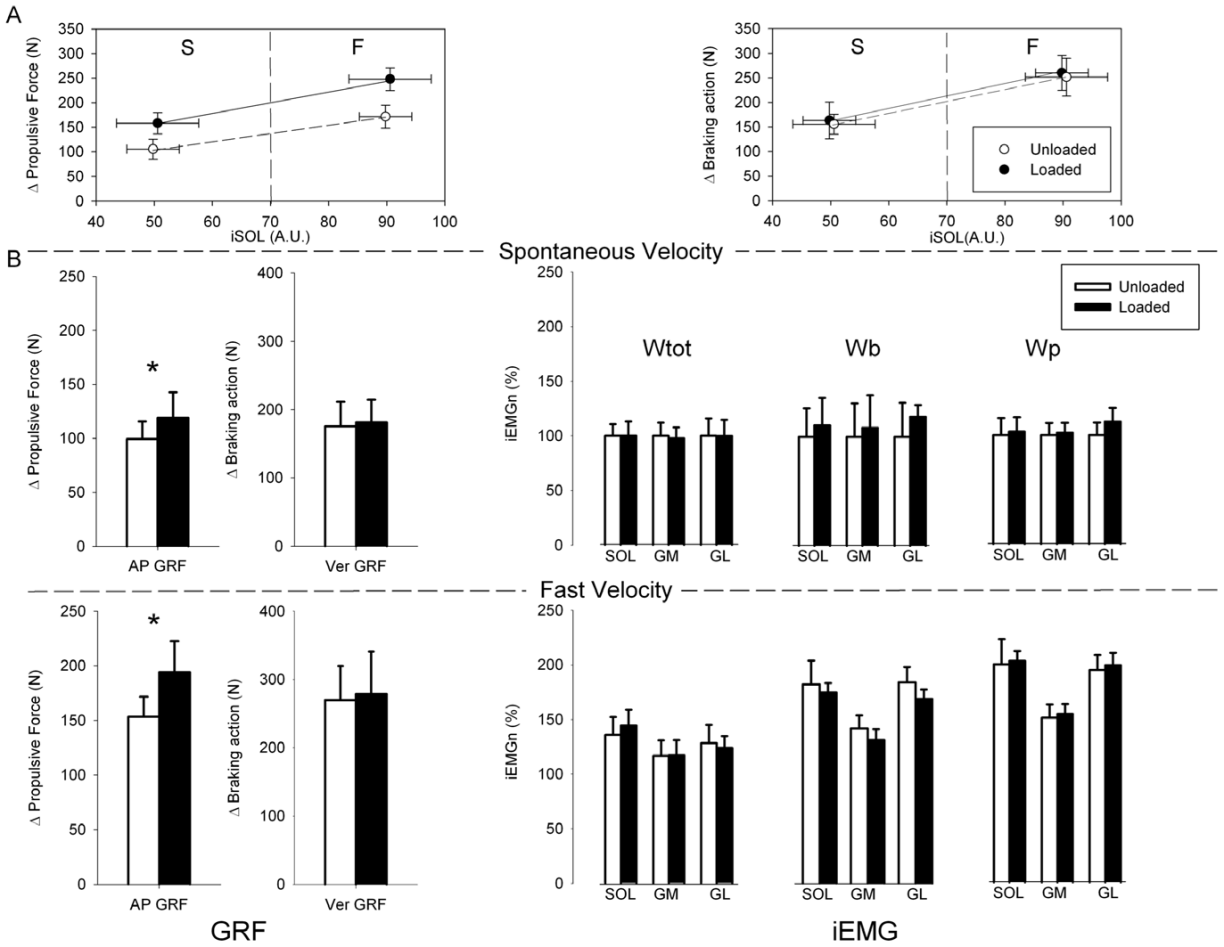
Divergent effects of load and walking velocity on propulsive force, braking action and triceps activity. The upper part of the Figure (A) contains two graphs, reporting the mean data from all trials of a representative subject. The left part of the left graph (spontaneous velocity, S) shows that AP GRF (the propulsive force) is larger when load is added (filled circle) compared to no-load (open circle). Notably, this increase occurs without changes in the SOL activity (measured during Wtot). A similar pattern is shown in the right part of the same graph (fast velocity, F). Note that F velocity is associated with an increase in SOL activity with respect to S velocity (abscissa): adding the load increases the propulsive force but does not further increase the amplitude of the burst. The lower part (B) contains two composite panels that summarize the results from all subjects. The left panel shows the mean and standard deviation of AP and Ver GRF, for the spontaneous (top) and fast velocity (bottom). Open bars refer to no-load, filled bars to added-load condition. The right panel shows the muscle activities for spontaneous (top) and fast velocity (bottom) conditions, unload and loaded, calculated within each time window (Wtot: entire burst, Wb: braking action, Wp: propulsive force). The EMG is expressed in percentage of the mean value recorded in the normal unloaded condition. Asterisks indicate p<0.05. There is an effect of velocity on braking action, propulsive force and muscle activity (all bars are higher in the bottom graphs), but no effect of load on any variable, except propulsive force (at both velocities).

Fig. 3 A shows the increase of the propulsive force (left panel) and of the braking action measured from the Ver GRF trace (right panel) plotted versus the mean SOL EMG level for one subject, for spontaneous and fast velocity. The values pertain to the time-window Wtot, from the onset to the termination of the EMG. The left panel indicates that the subject generated two different propulsive forces during the unloaded and loaded trials, while the corresponding mean EMG values were essentially the same for the same walking velocity. The right panel shows that neither the amplitude of the braking action nor the EMG activity were affected by load within each velocity. Hence, the extra load enhanced the AP GRF selectively. This is summarized in Fig. 3 B (left panel). The propulsive force increased on average by about 36% for the spontaneous velocity (F(1,9) = 101.1, p<0.001), and by about 29% for the fast velocity (F(1,9) = 8.1, p<0.05), showing that a significant increase in AP propulsive thrust was generated during the late stance by the added load. In contrast, the Ver GRF remained almost unchanged with the added load (F(1,9) = 0.11, p = 0.92) but increased significantly with velocity (F(1,9) = 5.7, p<0.05).

The right panel of Fig. 3 B shows the mean amplitude of the EMG activity of SOL, GM and GL recorded during the three time-windows (Wtot: entire burst; Wb: from onset of braking action until point 4 (as in Fig. 1); Wp: from onset of AP GRF increase until point 2). The graph has been built on the basis of the EMG data from the seven subjects in which we recorded SOL, GM and GL. Notably, also in the three subjects in which Sol was the sole muscle recorded from, SOL EMG behaved very much as depicted in this Figure. For the fast velocity condition, the amplitude of AP GRF, of the braking action and of the EMG increased compared to spontaneous velocity. However, within the same velocity, even when the amplitude of the propulsive force increased significantly as an effect of load, the grand mean of the EMG activity remained unchanged for SOL, GM and GL, regardless of the three time-windows used. Worth noting is that, when the subjects performed the fast walking trials, EMG activity increased concurrently with the increase in the braking action at fast velocity, but again there was no increase in EMG activity when the load was added. Remarkably, there were no changes with load even in the Wp interval corresponding to the propulsive phase of the stance period, in any muscle and for either velocity, i.e. when the active ‘push-off’ would be expected.

Therefore, it is fit to conclude that active recruitment of SOL, GM and GL muscle activity was not responsible for the increase in propulsive force required by the added load, but only for the increase in the braking action occurring with the increase in step length.

### Disequilibrium Torque

**Figure 4:**
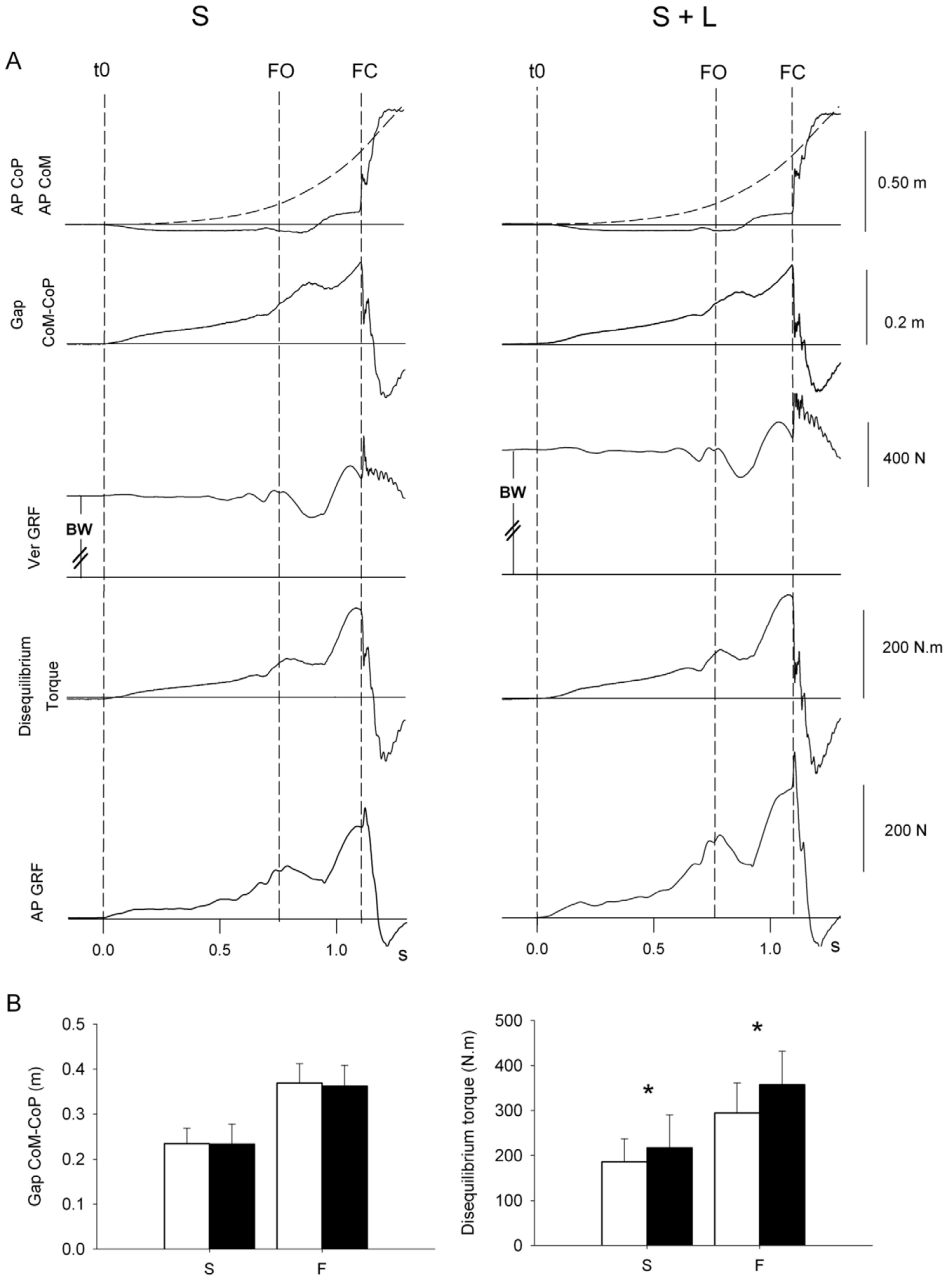
Disequilibrium torque calculation and its dependence on AP GRF. The upper traces in (A) show, from top to bottom, the time-course of the instantaneous AP position of CoP (the position of CoM is superimposed, dashed line), the CoM-CoP gap, the vertical GRF, the disequilibrium torque (calculated as the product of the gap by the vertical GRF), and the AP GRF. The traces are from one representative subject, during unloaded (left) and loaded condition (right) at spontaneous walking velocity. The bottom histograms (B) show the grand mean values (± SD) of gap (left graph) and torque (right graph), computed at FC for spontaneous (S) and fast velocity (F), unloaded (open bars) and loaded (filled bars) conditions. The gap increases with velocity, but does not change with load. Conversely, the torque increases with both velocity and load. Asterisks indicate p<0.05.

Fig. 4 A compares, in a representative subject, the time-course of the instantaneous antero-posterior positions of CoP (continuous trace) and of CoM (dashed trace), the CoM-CoP gap, the vertical GRF, the disequilibrium torque (the product of the gap by the vertical GRF) and the AP GRF, for the unloaded and loaded condition (left and right panels), at spontaneous velocity. Worth noting is the striking similarity of the traces of the disequilibrium torque and AP GRF between unloaded and loaded conditions. This similarity verifies the fact that the energy generated by CoM rotation around CoP is transformed into forward propulsive force. Visual inspection of the individual traces in Fig. 4 shows that load did not affect the CoM-CoP gap. However, both disequilibrium torque and AP GRF increased concurrently with load. To better understand this, the CoM-CoP gap and the disequilibrium torque at the instant of foot contact were calculated. The grand mean values and standard deviation of the gap and disequilibrium torque are reported in Fig. 4 B, in which gaps and torques are compared at spontaneous and fast velocities, unloaded and loaded conditions. The grand mean value of the gap measured at FC was the same between no-load and load conditions, at both spontaneous (F(1,9) = 0.0002, p = 0.98) and fast velocity (F(1,9) = 1.74, p = 0.22). At foot contact, gap was found to be around 49% ±4 of the step length and 48% ±4 for the S and S+L conditions, and 52%±4 and 51%±5 for the F and F+L conditions, respectively. The grand mean value of the torque was significantly larger under the loaded condition, both for the spontaneous (F(1,9) = 9.92, p<0.05) and for the fast velocity (F(1,9) = 163.98, p<0.001).

The disequilibrium torque depends on both CoM-CoP gap and Ver GRF. Since the CoM-CoP gap remained unchanged, the increase in torque solely depended on the changes in Ver GRF. In turn, the Ver GRF is composed of body weight and variations with respect to it due to the vertical acceleration of CoM. Since these variations (CoM fall and braking action) remained constant, then the increase in the disequilibrium torque was due to the absolute increase in body weight (4^th^ trace in Fig. 4 A, left & right) when subjects were loaded.

## Discussion

Our findings are not in keeping with the notion that the triceps surae is responsible for the generation of propulsive forces for walking. They rather show that the triceps supports the body while it translates over the ankle joint, restraining it from falling. Indirectly, though, triceps surae activity controls step length and walking speed.

### Rationale of the Investigation

To challenge the push-off hypothesis we have used the gait initiation paradigm and compared the condition, in which the subject was loaded, to that without load while maintaining the same progression velocity between experimental conditions. Since it has been repeatedly postulated that the triceps surae muscle is responsible for the ‘push-off’ action in the second part of the single stance phase of waking, we measured both biomechanical variables and the activity of soleus (SOL), gastrocnemius medialis (GM) and lateralis (GL) during the whole single stance period, to check whether any of these muscles, or all together, are accountable for the push-off.

The gait initiation paradigm should militate against our initial hypothesis (no active ‘push-off’) because the subject is initially motionless. A thrust to the ground support would seem necessary to generate a forward-propulsive force to propel the body forward, and even more so with the added load. This push-off would necessarily come from ankle plantar-flexor force, because plantar flexors are suitably arranged and are active during single stance [18], [23], [30]. Since laws of motion dictate that more force is required to propel a heavier object, we added a weight to the subject to induce an increase in the propulsive force. The significant increase of antero-posterior ground reaction force (AP GRF) that we observed during the loaded trials was indeed in accordance with the predicted effect of load.

Because of the linear relationships between progression velocity and triceps surae EMG activity, and between progression velocity and propulsive force [1], [31], the effect of velocity had to be isolated in order to unambiguously assert that triceps activity is or is not responsible of AP propulsive force generation. Thus, recording the triceps activity while imposing the same progression velocity between unloaded and loaded conditions permitted to extricate the postural from the propulsive role of the triceps.

Further, the effect of load was tested at two velocities. Replicating the effect of load at different velocity conditions corroborated the strength of the results. Our findings were consistent within the two velocity conditions, where GRF, kinematics and EMG are modified by the effect of speed. Therefore, we feel confident that our conclusions can be generalized to a range of walking velocities.

### The Role of the Triceps Surae in Push-off can be Rejected

GRF data alone undermine the push-off hypothesis. If the subjects were to increase their push off the ground to propel themselves when a load was added, then basically both the AP and Ver components of the force vector measured by the force platform should increase concurrently. However, only the amplitude of the rise in AP GRF, but not the amplitude of the braking action, increased when load was added.

Adding the load increased significantly the AP propulsive force throughout the entire gait initiation process including the preparatory and single-support stance phases, in the same proportion as the added load with respect to the body weight. In contrast, EMG activity of the triceps of the stance leg during the whole single-support phase, as well as within the time-interval corresponding to the vertical braking phase, did not change when the load was added. Therefore, the increase in the propulsive force, when a load was added to the walking subject, was not accompanied by any increase in triceps activity. In other words, the triceps does not participate in the augmentation of the AP component of the GRF (the propulsive force) connected with the increased body weight. Thus, our subjects need not push-off the ground to propel themselves forward.

The onset of the braking action was the first measurable mechanical event picked up by the force platform shortly after the onset of the triceps surae EMG activity. The time-interval between onset of EMG and onset of braking action was independent of the exact time of the braking action onset with respect to FO. This suggests a strong relationship between triceps surae contraction and braking action. AP GRF variations is instead linked to the CoM-CoP gap that builds up during the single-stance phase. Notably, the EMG did not even change within the time-window corresponding to the propulsive phase (Wp). These data therefore show that the triceps surae is responsible for the braking action seen in Ver GRF and that propulsive force is produced by the disequilibrium torque that is generated as CoM-CoP gap becomes more prominent during stance phase. This interpretation is in line with Simon *et al.* [10] and Sutherland *et al.* [3], who found that speed at late stance increased when ankle plantar-flexors were paralysed. Moreover, this behaviour is common to each of the three head of the triceps muscle. Neither SOL nor GM nor GL activity augmented their pattern of activity, commonly or independently, on increasing body weight. Therefore, in spite of their potentially independent activation during postural tasks [32], we consider safe to deduce that these three muscles are driven by a single motor program during gait, and all of them are devoted to body support.

In contrast, the triceps can increase its activity, and it did so at higher walking speed, in keeping with the data in the literature showing a relationship between velocity and triceps EMG [1]. When our subjects passed from the spontaneous to the fast speed, the GRF along both AP and Ver axes was enhanced along with the triceps surae EMG activity, but again EMG activity did not further increase on adding the load, in any examined time-window. On increasing velocity, EMG activity was slightly higher for SOL and GL than for GM. This simple observation lessens the risk that cross-talk between muscles can have blurred differences in their activation pattern; however it raises the question whether GM has an activation ceiling during locomotion.

Others have conducted experiments on a treadmill where an external force was applied to the subjects in order to manipulate the propulsive force. Stephens and Yang [33] loaded the subjects by adding a mass and unloaded them by means of winch and cable system anchored over their heads. They reported an augmentation of level and duration of SOL activity in the loaded condition, whereas unloading affected EMG duration only but not amplitude. Gottschall and Kram [34] used an aiding horizontal pulling apparatus connected at the waist of the subjects in order to decrease propulsion forces. They reported a decrease in GM but not in SOL activity and claimed that GM is involved in the generation of the propulsion force while SOL only provides body support. On the other hand, the horizontal force exerted by the pull may have changed body posture inclination with respect to the feet, as may happen with walking up a slope. This probably alters triceps surae function more into propulsion. Furthermore, McGowan *et al.* [35] argued that a horizontal pulling apparatus would disturb the ankle torque and adopted a protocol similar to that of Stephen and Yang [33], but allowed the vertical apparatus to slide over the subjects’ head. They also tested the effect of body mass to increase inertia by adding a load and pulling vertically in order to unload the subjects. Only SOL activity was affected by the change in body inertia, while both GM and SOL activity increased when body weight increased. Strikingly, GM and SOL activity decreased when subjects were unloaded, in opposition to what Stephens and Yang [33] found with almost the same protocol. Lewek [36] unloaded subjects as well at different speeds and found that SOL, GM and GL activity was affected by walking velocity but not by the unloading factor. The reasons behind the discrepancy between our experiment and those mentioned previousl*y* could be the non-negligible differences in kinematic and kinetic variables between over ground gait and treadmill walking [37], [38], [39]. This issue requires further investigation. Furthermore, the friction force (even qualified as low friction) and/or the inertia of the pulling apparatus used during those experiments could have altered the sensori-motor organization, in turn modifying EMG activity [40], [41]. Also, adding a load can alter the body CoM position depending on the placement of the added mass, and therefore the pattern of muscle activity. Since in our case the added load was positioned both anterior and posterior to the CoM position, the changes in body CoM position should be negligible. McGowan *et al.* [35] pointed out that a certain external force would alter joint moments when a lever arm is created between the force and the joint at hand. Therefore, adding an external force, in the experiments where a pulling apparatus was used, could have altered the behaviour of the triceps surae working across the ankle. More importantly, it is hard to keep the vertical pulling apparatus perfectly vertical at all times: the varying tension in the cable could affect Ver GRF, which we believe is responsible for generating disequilibrium torque, and slightly alter ankle torque and consequently normal triceps surae activity. On the contrary, Lewek [36] found with a similar protocol (treadmill walking and pulling apparatus) that SOL activity did not change when reducing ankle torque by applying an upward vertical force to the subject. This is contradictory to the results obtained by McGowan *et al.* [*35*] on the basis of which SOL activity was expected to decrease. Lewek’s [36] findings are instead clearly complementary to ours, in that the change in ankle torque was only affected by gravity both when subjects were unloaded and when they were loaded, while in both cases the EMG activity remained constant.

### Source of AP Propulsive Force

Two postulates have been used for explaining the process of generation of the AP propulsive forces during free walking: i) the triceps surae ‘pushing off’ the ground or ii) the transformation of the potential energy into the forward kinetic energy [12], [42]. The first postulate is discarded by our data. Our results are instead in line with Cavagna and Franzetti [12] and Cavagna *et al.* [43], who showed that the AP propulsive force comes from the transformation of the kinetic energy of the fall of the CoM during the single support phase into propulsive energy, and give further insight into the nature of the parameter that is controlled in order to produce propulsive force according to the demand.

The body is in equilibrium when the vertical ground projection of the CoM and the CoP are confounded [15], [16]. During locomotion, the CoM and the CoP in the sagittal plane must be separated in order to create a lever arm and thus a disequilibrium torque driven by the force of gravity. In gait initiation, this is done initially by means of a backward shift of the CoP [22], [23], [29]. Then, during the single stance phase the body starts rotating around the ankle forcing the CoP to move forward. At late stance, the forward shift of the CoP is stopped as it is constrained within the anterior geometrical limit of the stance foot. Meanwhile, the CoM maintains its forward momentum and thus the lever arm between CoM and CoP cannot but sharply increase (Fig. 4).

Examination of the traces of CoM-CoP gap and of disequilibrium torque explains how the AP GRF is created. While the time courses of the AP GRF, AP CoM and CoP displacements are different, the time courses of the gap between CoM and CoP and of the disequilibrium torque are quite superimposed to that of AP GRF. In other words, AP propulsive force is modified by the unique control of the CoP position and the GRF vector. Similar results were obtained by Gruben and Boehm [44], [45], who showed that during level walking CoP is shifted such that the GRF vector always points to a specific reference in close proximity of CoM. In our case, when the subjects were loaded, the sole increase of their body weight was accountable for the increase in the disequilibrium torque that appears on Fig. 4, since gap or EMG activity were not affected by the load. This increase in torque was transformed into AP GRF according to Cavagna and Fanzetti [12].

The lever-arm was found to be around 48.5% and 51.5% of step length for spontaneous and fast steps respectively. Therefore, in order to increase CoM progression velocity (in the fast speed condition), one must increase step length, which will in turn increase CoM-CoP gap because a longer step implies an ampler displacement of CoM. Since CoM-CoP gap increases with a lengthier step then so will the disequilibrium torque along with the propulsive force. However, to obtain a larger CoM-CoP gap, forward momentum of the CoM should increase, while the CoM vertical position has to be kept from descending beyond a certain threshold, or else lifting up CoM again to perform the second step would not be energy efficient. To do so, subject have to increase the braking action, which requires more force and motor units to be recruited in the three muscles SOL, GM and GL. This increase of EMG activity to provide a stronger body support at higher velocity is in line with the results provided by Liu *et al.* [19].

### The Braking Action of the Triceps Surae

EMG activity of the triceps surae remained unchanged when increasing propulsive force (as needed by adding an extra load to body weight) while maintaining the same velocity. This opens the question of the motor strategy used to control balance during gait. Some authors used different modelling techniques and agreed that the triceps surae does play a role in body support, a term broadly used to designate the control of balance [6], [18], [19], [46]. Body support was defined as a vertical force applied by the triceps surae to resist gravity or, in other terms, to brake the fall of the CoM. Vertical braking of the CoM has been previously investigated in elderly people and in subjects suffering from motor disorders such as Parkinsonian patients. Interestingly, both populations showed either insufficient or no braking of the CoM when they initiated gait [13], [47]. Here, the braking of CoM fall is seen clearly in the Ver GRF profile, since GRF increased beyond body weight when the downward velocity of CoM reversed, resulting in actively deceleration of the CoM before foot contact (Fig. 1). Furthermore, when the load was added, the Ver GRF recorded throughout the trial increased systematically by around 200 N, as predicted. However, for the same velocity, the vertical braking action (the distance 3 to 4 in the Ver GRF traces of Fig. 1) was not affected by load and remained constant (bars in Fig. 3 B, left panel). This can be explained by ideally considering the body as an inverted pendulum, where the body is a point mass positioned at CoM and rotates around CoP. Newton’s law of motion τ_ext_ = I·α (where τ_ext_ is the net external torque, I is the moment of inertia and α is the angular acceleration) can be then applied. In our case, the equation is expressed as mg·(CoM-CoP gap) = m·r^2^α (where m is body mass, g is the gravitational acceleration and r is the distance between CoM and CoP). By dividing both parts of the equation by m results in the downward acceleration of the CoM being only affected by the CoM-CoP gap, which should be constant for the same step length according to Gruben and Boehm [44], [45], regardless of the load.

Thus, by controlling CoM-CoP gap, the triceps muscle exerts the activity necessary for keeping the body upright in spite of the added load. Even under standing condition, a torque is exerted by triceps surae in order to counteract the gravity torque, since the CoM projection lies just in front of the ankle joint [14], [15]; the added load requires a minor but definite increase in the antigravity activity. At gait initiation, in the loaded compared to unloaded condition, subjects anticipated the onset of the burst in the three triceps muscles (SOL, GM and GL). This low-level, early activation of the triceps surae muscle, performing eccentric contraction, would help stiffening and stabilising the ankle joint as a result of the increased bodyweight, right at the critical time when the double support turns into the single support, and when the foot arch flattens under the effect of the new load on the single supporting foot and of the forward tilt of the tibia [48].

On the other hand, when subjects performed fast walking, the braking action augmented, since more force was required to counter the larger CoM vertical displacement. The increase in vertical action was accompanied by an increase in triceps EMG activity. The reason behind the increased EMG during the braking action phase is to prevent the CoM from falling beyond a certain level, where rising it up again for the second step would be metabolically costly as energy transfer would be less effective. On the other hand, velocity *per se* had no significant effect on the time of onset of triceps muscles’ burst. Our results are complementary to those of Holt [49], who found that the vertical displacement of CoM was increased with faster walking velocity but was not affected by load. Interestingly, triceps activity is maintained for some time after FC and is silenced briefly prior to lifting of the lagging leg into swing as the tibialis anterior of that leg becomes active. Kuo [50] has explained that in double stance CoM does not require to be propelled and lifted, but redirected due to the net action of both legs exerting positive (lagging leg) and negative (leading leg) work, since CoM ends single support with an appropriate height, momentum and energy. However, it is also possible that the work performed by the triceps surae of the lagging leg during double stance contributes to lifting that leg into swing along with hip flexor activity [6].

Other evidences from remote lines of investigation point to the triceps as a major controller of the effects of gravity on body mass. Sinkjaer *et al.* [51] applied slow-velocity enhancements and reductions to the natural ankle dorsiflexion during the stance phase of walking, thus mimicking variations potential changes in the ankle dorsiflexion trajectory connected to uneven ground during walking, and found that dorsiflexion enhancements generated gradual increments in the soleus EMG. Afferent feedback from large- and medium-diameter spindle sensory fibres was shown to contribute to the background SOL activity [52], [53]. Such mechanism may be at least in part responsible for the gradual build-up in the SOL EMG burst during the stance phase of gait initiation and contribute to the control of the braking action, as well as for the increase in triceps activity during fast walking (accompanied by larger ankle dorsiflexion). On a different vein, it is pertinent to recall here that by merely controlling the braking action trough triceps activity, the CNS can modulate walking velocity, and parsimoniously produce the difference in step length between legs necessary for producing steering of the walking trajectory [54], [55].

### Summary

Triceps surae EMG activity did not change when propulsive forward force increased due to the effect of adding a load. This was true both for spontaneous and for fast velocity condition. Therefore the hypothesis stating that the triceps pushes-off the ground to generate propulsive force is discarded. The triceps is instead responsible for balance control by braking CoM vertical displacement. In this light, the ‘push-off’ term itself is more a confounding misnomer than a short designation of the real increase in propulsive torque occurring in the second part of the stance phase. The forward progression of the body is only due to the transformation of the potential energy of the CoM fall into forward kinetic energy [42]. EMG activity increases only when the vertical braking action of CoM augments as a result of increasing body support demand while walking quickly. By controlling body support, triceps modulates the antero-posterior distance between the centre of mass (CoM) and the centre of foot pressure (CoP), thereby indirectly modulating walking velocity.
